# Involvement of Microglia Activation in the Lead Induced Long-Term Potentiation Impairment

**DOI:** 10.1371/journal.pone.0043924

**Published:** 2012-08-31

**Authors:** Ming-Chao Liu, Xin-Qin Liu, Wen Wang, Xue-Feng Shen, Hong-Lei Che, Yan-Yan Guo, Ming-Gao Zhao, Jing-Yuan Chen, Wen-Jing Luo

**Affiliations:** 1 Department of Occupational & Environmental Health and the Ministry of Education Key Lab of Hazard Assessment and Control in Special Operational Environment, School of Public Health, Fourth Military Medical University, Xi'an, China; 2 Department of Anatomy, Histology and Embryology, K.K. Leung Brain Research Centre, Fourth Military Medical University, Xi'an, China; 3 Department of pharmacology, Fourth Military Medical University, Xi'an, China; Imperial College London, United Kingdom

## Abstract

Exposure of Lead (Pb), a known neurotoxicant, can impair spatial learning and memory probably via impairing the hippocampal long-term potentiation (LTP) as well as hippocampal neuronal injury. Activation of hippocampal microglia also impairs spatial learning and memory. Thus, we raised the hypothesis that activation of microglia is involved in the Pb exposure induced hippocampal LTP impairment and neuronal injury. To test this hypothesis and clarify its underlying mechanisms, we investigated the Pb-exposure on the microglia activation, cytokine release, hippocampal LTP level as well as neuronal injury in *in vivo* or *in vitro* model. The changes of these parameters were also observed after pretreatment with minocycline, a microglia activation inhibitor. Long-term low dose Pb exposure (100 ppm for 8 weeks) caused significant reduction of LTP in acute slice preparations, meanwhile, such treatment also significantly increased hippocampal microglia activation as well as neuronal injury. *In vitro* Pb-exposure also induced significantly increase of microglia activation, up-regulate the release of cytokines including tumor necrosis factor-alpha (TNF-α), interleukin-1β (IL-1β) and inducible nitric oxide synthase (iNOS) in microglia culture alone as well as neuronal injury in the co-culture with hippocampal neurons. Inhibiting the microglia activation with minocycline significantly reversed the above-mentioned Pb-exposure induced changes. Our results showed that Pb can cause microglia activation, which can up-regulate the level of IL-1β, TNF-α and iNOS, these proinflammatory factors may cause hippocampal neuronal injury as well as LTP deficits.

## Introduction

Lead (Pb) is known to cause irreversible neurological disturbances [1]. Environmental low-level Pb caused neurotoxic impairment is a severe hazard worldwidely [2], [3]. Extensive studies have shown the effect of low-level Pb exposure on growth, development and cognitive function [4], [5]. These extensive studies revealed that Pb exposure at low doses is extremely dangerous and can cause learning and memory impairments [6]. The hippocampus is a brain center for learning and memory [7]. LTP of the hippocampal excitatory synaptic transmission is thought to be a pattern of manifestation for synaptic plasticity [8] which is believed to be the underlying mechanisms for hippocampus-dependent learning and memory [9].

On the other hand, the contribution of microglia to the hippocampus dependent learning and memory becomes the research focus recently. Microglia activation can impair the learning and memory via released interleukin (IL)-1 [10]. Inhibiting the microglia activation can rescue the learning and memory deficits in a murine model of human immunodeficiency virus (HIV) type 1 encephalitis [11]. Meanwhile, the microglia activation is suggested to be involved in some form of LTP [12] and such effect may be mediated by the adenosine receptor P2X7 located on the microglias [13]. Although one previous study suggested that the microglia released IL-8 is involved in the inhibition of hippocampal LTP [14], whether and how microglia contributes to hippocampal LTP remains unclear.

Our pilot study revealed that low-level Pb-exposure induced hippocampus-dependent learning and memory deficits as well as activation of microglia. Microglia may contribute to the hippocampal LTP which is recognized as one mechanism for learning and memory. Thus, we raised the hypothesis that activation of microglia is involved in the Pb exposure induced hippocampal LTP impairment, neuronal injury and learning and memory deficits. To test this hypothesis and clarify its underlying mechanisms, we investigated the Pb-exposure on the microglia activation, cytokine release, hippocampal LTP level as well as neuronal injury in adult Sprague-Dawley rats (SD-rats) or primary hippocampal and microglial cell cultures. The involvement of microglia activation in the Pb induced LTP impairment was also confirmed by inhibiting microglia activation with minocycline, a microglia inhibitor.

## Materials and Methods

### Materials

Cell culture ingredients were purchased from Invitrogen (Grand Island, NY, USA). D-Hanks solution was bought from GiBCO (Grand Island, NY, USA). Recombinant human granulocyte-macrophage colony-stimulating factor (GM-CSF) and poly-L-lysine were obtained from SIGMA (USA). Transwell co-culture plates and other cell culture equipment were bought from Costar (Corning, NY, USA). SD rats were obtained from the Animal Experiment Center of the Fourth Military Medical University. Analytical pure lead acetate was purchased from SIGMA (USA). Minocycline was bought from SIGMA (USA). Polyclonal mouse anti-OX42 antibody, and polyclonal mouse anti-NeuN antibody were from CHEMICON (Hampshire, UK). The fluorescence secondary antibodies were purchased from Vector Laboratories (Burlingame, CA, USA). TNF-α and IL-1β Enzyme-linked immunosorbent (ELISA) kits were purchased from eBioscience (San Diego, CA, USA). Lactate dehydrogenase (LDH)-cytotoxicity assay kits were purchased from Biovision (Biovision Inc, USA). All other reagents were purchased from SIGMA (USA). The inverted microscope, the fluorescence microscope and the Laser confocal microscope were bought from OLYMPUS (Tokyo, Japan). The enzyme linked immunosorbent spectrophotometer was obtained from SHIMADZU (Nakagyo-ku, Kyoto, Japan).

### Animals and treatments

All procedures involving animals were carried out in strict accordance with the international standards of animal care guidelines (guide for the care and use of laboratory animals) and were approved by the institutional animal care and use committee of Fourth Military Medical University (Permit Number:12001). Male SD rats were obtained from the Animal Experiment Center of the Fourth Military Medical University. The animals were maintained in a 12/12 light/dark cycle and a temperature-controlled room, with food and water available ad libitum. The rats were assigned to four groups (20 for each group): control group, minocycline treated group, Pb treated group, Pb and minocycline treated group. The animals were fed with lab chow pellets (obtained from the Animal Experiment Center of Fourth Military Medical University). On the third day after arrival (aged 22–24 days), the animals were exposed to Pb *via* drinking water. Lead acetate (SIGMA, USA) was dissolved in distilled water with a concentration of either 0 or 100 ppm [15]. Pb concentrations were verified using electrothermal atomization atomic absorption spectroscopy (AAS). Rats were exposed to Pb from 24 to 80 days of age. Minocycline (120 mg/kg per day in 5% sucrose; SIGMA, USA) was delivered via oral gavage [16] in Pb expose 0 or 100 ppm rats. Water consumption was monitored every two days and individual body weight was measured weekly during the experimental period.

### Primary cultures and cell stimulation

#### 1. Hippocampal neuronal cultures

Rat primary hippocampal cultures were prepared following a previously described protocol [17], [18], [19] with some modifications. Briefly, hippocampal tissues were dissected from postnatal day 1 SD rats and dissociated mechanically without using enzymes. Cells were seeded in 12-well (6×10^7^/ml) culture plates pre-coated with poly-L-lysine (20 µg/ml) and maintained in 0.5 ml/well of Dulbecco's Modified Eagle Medium: Nutrient Mixture F-12 (Ham) (1∶1) (DMEM/F12) supplemented with 10% heat-inactivated fetal calf serum (FCS), 1 g/L glucose, 2 mmol L-glutamine, 100 U/ml penicillin, and 100 U/ml streptomycin. Cultures were maintained at 37°C in a humidified atmosphere of 5% CO_2_ and 95% air. Cultures were replenished with 0.5 ml/well fresh medium 3 days later and were used for treatment 3 days later.

#### 2. Microglia cultures

Microglia cells were derived from postnatal day 1 SD rat brains. Cerebro-cortices were isolated and trypsinized. Cortices were dissociated mechanically and plated into tissue culture T-75 flasks in DMEM/F12 with L-glutamine (Invitrogen, Carlsbad, CA, USA) containing 15% heat-inactivated FCS and 20 µg/L GM-CSF. After 24 h all media and tissue were removed and replaced with fresh media. After 7 days, one half of the media was replaced and cells were maintained in a mixed glial culture until day 14. At 14 days *in vitro*, microglia were removed from the mixed glial culture *via* a rotating shaker at 60 rpm for 20 h, the supernatant was collected and seeded in 12-well culture plates and cultured as above.

#### 3. Cell stimulation

For the microglia activation assay, the purified microglia were seeded in 12 well culture plates, cultured in 0.5 ml/well of DMEM/F12 containing 10% FCS, 2 mmol/L-glutamine, 100 U/ml penicillin, and 100 U/ml streptomycin. Cells were treated with 50 µmol/L Pb for 48 h.

For the neuroprotective effects assay, the hippocampal neurons were seeded and cultured in the bottom of Transwell plate, the purified microglia were seeded on the semipermeable membrane of Transwell insert, then put the Transwell insert into a normal 12 well culture plate, cultured and treated with 50 µmol/L Pb and 45 µmol/L minocycline for 48 h. The inserts were removed from the normal 12 well culture plate and immediately co-cultured with hippocampal neuronal cultures for 48 h in Transwell plates.

For cytotoxicity detection, the culture medium of each group were removed for LDH release assay using a LDH-cytotoxicity assay kit (Biovision Inc, USA) according to the manufacturer's protocol. The relative absorbance of all samples was measured at 490 nm. The measurement was repeated three times at a 5-s interval and the numbers of each group were calculated with the following formula: Cytotoxicity = (A test sample-A blank control)/(A positive control- A blank control)×100%.

### Determination of Pb concentrations in blood and hippocampal samples

At the end of the study, six animals from each group were decapitated. Blood samples were collected into heparinized syringes and used to determine blood Pb using AAS. Brain tissues were dissected and the hippocampus was quickly dissected on ice and used to determine Pb using an electrothermal atomization AAS.

### Long-term potentiation (LTP) recording in hippocampal slices

#### 1. Slice preparation

Coronal brain slices (300 µm) from a 6 to 8 week-old SD rat, containing the hippocampus, were prepared using standard methods [20]. Slices were transferred to a submerged recovery chamber containing oxygenated (95% O_2_ and 5% CO_2_) artificial cerebrospinal fluid (ACSF) (124 mM NaCl, 4.4 mM KCl, 2 mM CaCl_2_, 1 mM MgSO_4_, 25 mM NaHCO_3_, 1 mM NaH_2_PO_4_, and 10 mM glucose) at room temperature for at least 1 h.

#### 2. Whole-cell recordings

Experiments were performed in a recording chamber on the stage of an Axioskop 2 FS microscope with infrared differential interference contrast (DIC) optics for visualizing whole-cell patch-clamp recordings. Excitatory postsynaptic currents (EPSCs) were recorded from pyramidal neurons in the CA1 region using an Axon 200B amplifier (Axon Instruments, CA, USA), and stimulations were delivered using a bipolar tungsten stimulating electrode which was placed on Schaffer collateral-commissural fibers in the CA3 stratum radiatum. Alpha-Amino-3-hydroxy-5-methyl-4-isoxazolepropionic Acid (AMPA) receptor-mediated EPSCs were induced by repetitive stimulations at 0.02 Hz and neurons were voltage clamped at −70 mV. After obtaining stable EPSCs for at least 10 min, LTP was induced by 80 pulses at 2 Hz paired with postsynaptic depolarization at +30 mV (we called pairing training). The recording pipettes (3–5 MΩ) were filled with solution containing (mM) 145 K-gluconate, 5 NaCl, 1 MgCl_2_, 0.2 EGTA, 10 HEPES, 2 Mg-ATP, and 0.1 Na_3_-GTP (adjusted to pH 7.2 with KOH). Picrotoxin (100 µM) was always present to block gamma-aminobutyric acid (GABA) A receptor-mediated inhibitory synaptic currents. Access resistance was 15–30 MΩ and monitored throughout the experiment. Data were discarded if access resistance changed more than 15% during an experiment. Results are expressed as means ± SEM. Statistical comparisons were performed using the Student's t test.

### Immunocytochemistry

Neurons were detected with anti-NeuN antibody and microglial cells were detected with anti-OX42 antibody. Briefly, cells from primary culture were fixed with 3.7% paraformaldehyde for 30 min and cells from primary culture or slices form cryopreserved tissue were followed by blocking for 1 h with phosphate buffered saline (PBS) containing 0.4% Triton X-100, 2% bovine serum albumin (BSA) and 3% normal goat serum. After blocking, cells or slices were incubated with primary antibody overnight at 4°C. Cells or slices were then washed with PBS and incubated for 1 h with the secondary antibodies (anti-rabbit/or goat-FITC and antimouse-Rhodamine, Jackson ImmunoResearch, West Grove, PA, USA) at room temperature and then rinsing with PBS buffer. In *in vitro* experiments, neurons were stained with anti-NeuN antibody and then the apoptotic neurons were labeled *in situ* using the TUNEL. The rate of cell apoptosis was counted apoptotic cells and total neurons from 8–9 random fields of a coverslip and calculates the rate of cell apoptosis. In *in vivo* experiments, neurons were stained with anti-NeuN antibody and then the apoptotic cells were labeled *in situ* using the TUNEL. The number of apoptotic cells was counted in DG zone of hippocampus. Layers were determined according to anatomical criteria [21]. The rates of activated microglia were evaluated by counting the number of activated microglial cells from OX42-immunoreactive cells in DG zone of hippocampus or from 8–9 random fields of a coverslip. Expression of iNOS was evaluated by measure the average fluorescence of iNOS-immunoreactive cells. Cells were examined and recorded under an OLYMPUS BX51 fluorescent microscope equipped with DP-BSW software (OLYMPUS, Japan).

### Quantitation of secreted TNF-α and IL-1β

TNF-α and IL-1β in the culture supernatant were detected by an ELISA kit (eBIOSCIENCE). Briefly, the 96 well culture plates were coated with coating buffer at 4°C overnight, the plates were rinsed five times with wash buffer, and the plates were blocked with 1× Assay Diluent and incubated for 1 h at room temperature. The standard preparation was diluted with Assay Diluent and 100 µl of the standard preparation was added to the wells, at the same time, 100 µl of the sample was added to the wells, the plate was covered and incubated for 2 h at room temperature. The plate was then rinsed as before and 100 µl of the detecting antibody was added to the wells and incubated for 1 h at room temperature, then rinsed as before. 100 µl of Avidin-HRP was added to each well and incubated for 30 min at room temperature, the plate was rinsed 7 times, 100 µl of base solution was added to each well and incubated for 15 min at room temperature, then 50 µl of stop solution was added to each well and the plate was read at 450 nm.

### Statistical analysis

Data are expressed as the mean ± standard deviation (SD) except specified and analyzed with one-way analysis of variance (ANOVA) followed by a least significant-difference test for multiple comparisons where appropriate. A *P* value less than 0.05 was considered as statistically significant.

## Results

### Blood and hippocampal Pb level, body weight and water consumption following Pb exposure

Exposure to Pb in drinking water under the current dose regimen (100 ppm) resulted in a 4.5-fold increase in blood Pb (BPb) as compared to control rats (*p*<0.05) (Fig. S1). Similar to changes in BPb, Pb exposure resulted in 1.26-fold increases in Pb concentrations among hippocampus compared to control rats (*p*<0.05) (Fig. S2). Results also showed there are no significant differences between control and Pb groups (*P*>0.05) in body weight (Fig. S3) and water consumption (Fig. S4). This result demonstrates that 100 ppm Pb in drinking water didn't significantly affect the diet and growth of rats.

### Pb treatment reduced hippocampal LTP levels

**Figure 1:**
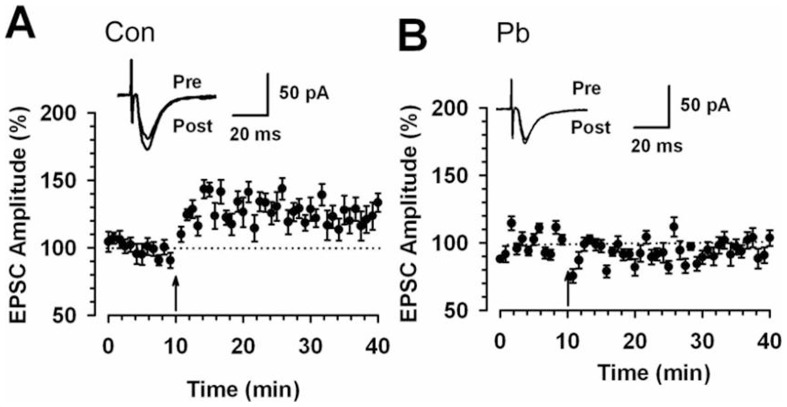
Abolishment of hippocampus synaptic potentiation in 100 ppm Pb treated rats. (A) LTP was induced in hippocampal pyramidal neurons in control rats (n = 13 slices/6 rats, t-test; *P*<0.001 compared with baseline). (B) LTP was lost in hippocampal pyramidal neurons in Pb treated rats (n = 8 slices/6 rats, t-test; *P*>0.05 compared with baseline). Pairing training is indicated by an arrow. The dashed line indicates the mean basal synaptic responses.

Previous reports have shown that Pb triggers a series of neuronal injuries and causes learning and memory deficits [22], [23], [24]. LTP is one important mechanism underlying hipopocampal learning and memory [7]. Thus, it's important to investigate the effect of Pb on hippocampal LTP. To determine how Pb affects hippocampus LTP, we used an *in vivo* approach using a Pb poisoning model in the present study. Using *ex vivo* slice preparations derived from the CA1 region of the rat hippocampus, we first assessed the impact of Pb on synaptic plasticity. We used the typical LTP induction paradigm to trigger LTP in hippocampal slices [25]. The results show that pairing training induced a significant, long-lasting potentiation of synaptic responses in slices from control rats (mean 129.6±7.5% of baseline, n = 13 slices/6 rats, t-test; *P*<0.001 compared with baseline responses, Fig. 1A). In contrast, synaptic potentiation was absent in slices from Pb treated rats (88.6±7.1%, n = 8 slices/6 rats, t-test; *P*>0.05 compared with baseline, Fig. 1B). These results show that Pb reduced the induction of LTP in the hippocampus of adult rats.

### *In vivo* Pb exposure activates microglia and induces hippocampal neuronal injury

**Figure 2:**
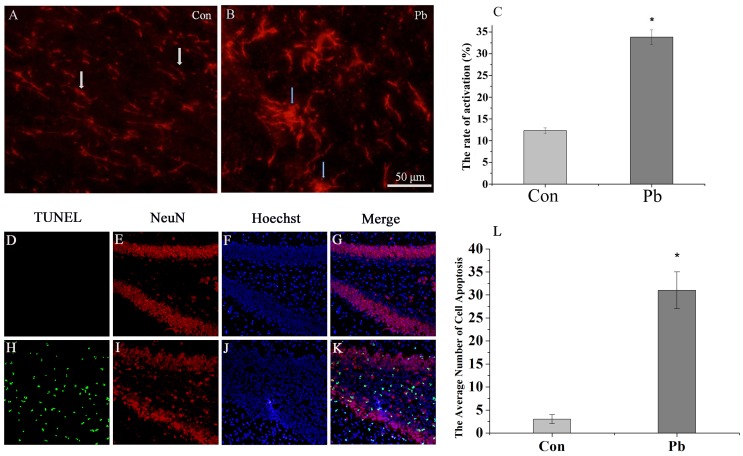
Effects of hippocampal neuronal injury and microglia activation induced by Pb in the hippocampus. Microglia activation was detected by immunocytochemistry (OX42). (A) control; (B) 100 ppm Pb for 8 weeks. Activation of microglia was evaluated by counting the number of activated microglial cells (B blue arrow); White arrows (A) indicate resting microglia. (C) The results were quantified and are expressed as the mean ± S.D. of average activated cell rate in random fields (n = 20). * *P*<0.05 compared with control groups. Scale bar indicates 50 µm. Hippocampal neuronal injury was detected with *in situ* TUNEL (green fluorescence). (K) The apoptotic neurons in the Pb group were significantly higher than the control group (G). Hippocampal neuronal injury was evaluated by analyze the number of apoptotic neurons of each group. (L) The results were quantified and are expressed as the mean ± S.D. of apoptotic neurons in DG zone of hippocampus (n = 8). * *P*<0.05 compared with control groups. Scale bar indicates 50 µm.

Recent studies have demonstrated that administration of lipopolysaccharide (LPS) can activate microglia and impair the level of learning and memory [26]. In this study, we subjected weaning rats to Pb. Analysis of the activation level of microglia from the hippocampus of weaning rats treated with Pb by immunocytochemistry, revealed microglia activation as evidenced by the key morphological changes including the thickening and retraction of branches, the increasing of cell body (33.69±3.48%, n = 20) (Fig. 2B and C) from the resting state compared with the control group (12.37±1.09%, n = 20) (Fig. 2A and C).

Studies have shown that treatment with Pb can cause hippocampal neuronal injury. In this study we also confirmed that Pb induced obvious neuronal apoptosis (31±4, n = 8) (Fig. 2H–K and L) in the hippocampus of weaning rats treated with Pb compared with the control group (3±1, n = 8, *P*<0.05 VS Pb group) (Fig. 2D–G and L).

### Pb pretreatment induced *in vitro* microglia activation

**Figure 3:**
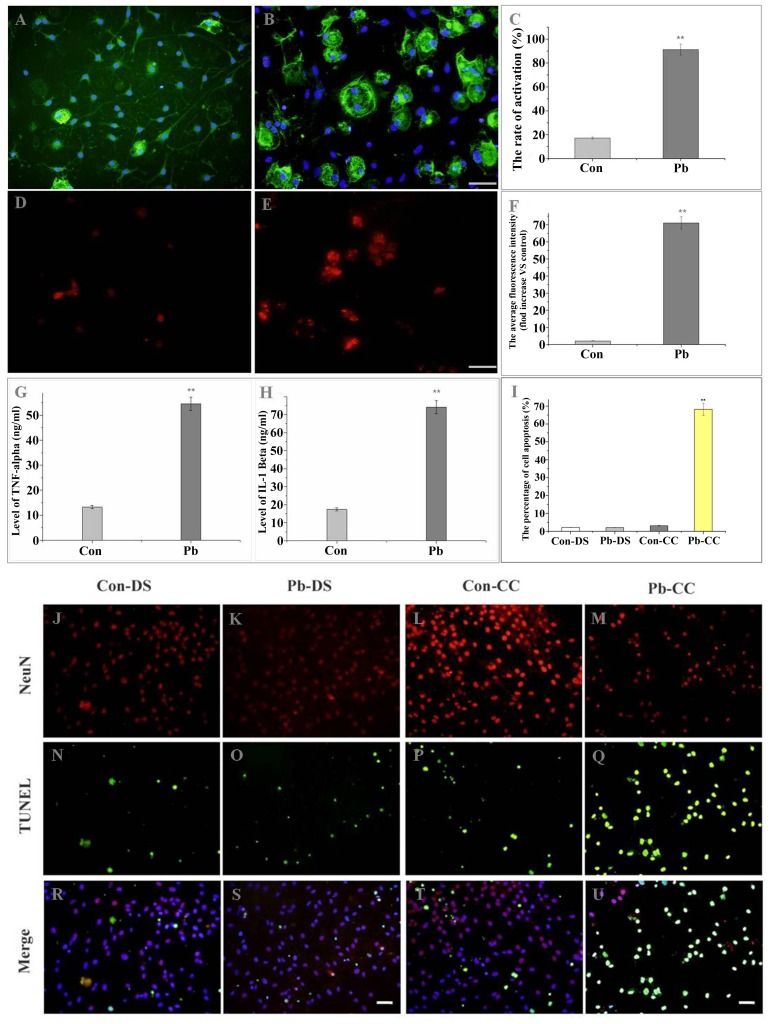
Pb activated microglia, enhanced TNF-α and IL-1β secretes, iNOS expression, and induces hippocampal neuronal injury. Purified microglia cells were seeded in 12-well culture plates. Cells were treated with vehicle (control) (A) or Pb (50 µmol) (B) for the indicated times. Microglia activation was detected by immunocytochemistry (OX42). (A) control; (B) 50 µmol Pb for 48 h. (C) The results were quantified. Results (A and B) are expressed as the mean ± S.D. of average activated cell rate in random fields (n = 20). **P<0.001 compared with control groups. Scale bar indicates 50 µm. iNOS was detected by immunofluorescence staining. Activation of iNOS was evaluated by measure the average fluorescence of iNOS-immunoreactive cells (D, E). (F) The results were quantified and are expressed as the mean ± S.D. of average fluorescence of iNOS-immunoreactive cells in random fields (n = 20). ** *P*<0.001 compared with control groups. Scale bar indicates 50 µm. TNF-α (G) and IL-1β (H) were determined by ELISA. Purified microglia were cultured with vehicle (control) or Pb (50 µmol) for 48 h for the TNF-α and IL-1β assay, respectively. ** *P*<0.001 compared with control groups. The purified microglia were co-cultured with hippocampal neurons for 48 h after culturing with vehicle (control) (L, P, T) or Pb (50 µmol) (M, Q, U) for 48 h. Apoptosis of hippocampal neurons was detected with *in situ* TUNEL (green fluorescence). Degeneration of hippocampal neurons was evaluated by counting the number of apoptotic neurons. As compared with the co-culture method, hippocampal neurons were also treated with vehicle (control) (J, N, R) or Pb (50 µmol) (K, O, S) directly for 48 h. (I) The results were quantified and are expressed as the mean ± SD of apoptotic neurons percentage in random fields (n = 20). ** *P*<0.001 compared with control groups. Scale bar indicates 50 µm. CC in figure means co-culture method; DS in figure means direct stimulation method.

To investigate if Pb induced microglia activation first and then followed with neuronal injury, we established an *in vitro* neuron-microglia co-culture model. In order to determine whether Pb exposure can activate microglia, we treated microglial cultures with 50 µmol/L Pb for 48 h based on MTT assay (Fig. S5). Immunocytochemistry showed that stimulated microglia were present as mononuclear macrophages (91.21±4.56%, n = 20) (Fig. 3B), and cells in the control group were mostly in the resting state (17.11±0.86%, n = 20) (Fig. 3A), which strongly indicated that Pb stimulation caused microglial activation (*P*<0.001 VS control) (Fig. 3C).

### Activated microglia cause hippocampal neuronal apoptosis *in vitro*

After treatment with Pb, microglial cells were co-cultured with hippocampal neurons. The effect of activated-microglia on hippocampal neuronal apoptosis in cultures was determined by TUNEL assay. As shown in Fig. 3, pretreatment of microglia with Pb significantly increased the number of apoptotic neurons from the hippocampal primary culture (Con: 3.11±0.16%, n = 20, Fig. 3 I, M, Q, U; Pb: 68.13±3.41%, n = 20, Fig. 3 I, L, P, T). In the group that Pb was applied directly to isolated neurons, the number of apoptotic neurons was not different from that of the control group (2.13±0.11%, n = 20) (Fig. 3 I, J, N and R) and the Pb-treated group (2.01±0.11%, n = 20) (Fig. 3 I, K, O and S). These results indicated that activated microglia caused hippocampal neuronal death. These findings raise the possibility that hippocampal neurons may be easily damaged by microglias which were activated with Pb.

### Inflammatory factors were up-regulated after stimulation with Pb *in vitro*

It has previously been reported that activated microglia secrete many different inflammatory factors [27], [28]. We found that after treatment with Pb, the expression of iNOS was at a significantly high level (71.08±3.55, n = 20) (Fig. 3 E and F) compared with control group (2.13±0.11, n = 20) (Fig. 3 D and F). We also found that TNF-α (Con: 13.18±0.54%, n = 6; Pb: 53.88±2.71%, n = 6) (Fig. 3 G) and IL-1β (Con: 16.84±0.63%, n = 6; Pb: 71.58±3.68%, n = 6) (Fig. 3 H) were induced after exposure to Pb.

### *In vitro* pretreatment with minocycline inhibits Pb induced upregulations of iNOS, TNF-α and IL-1β

**Figure 4:**
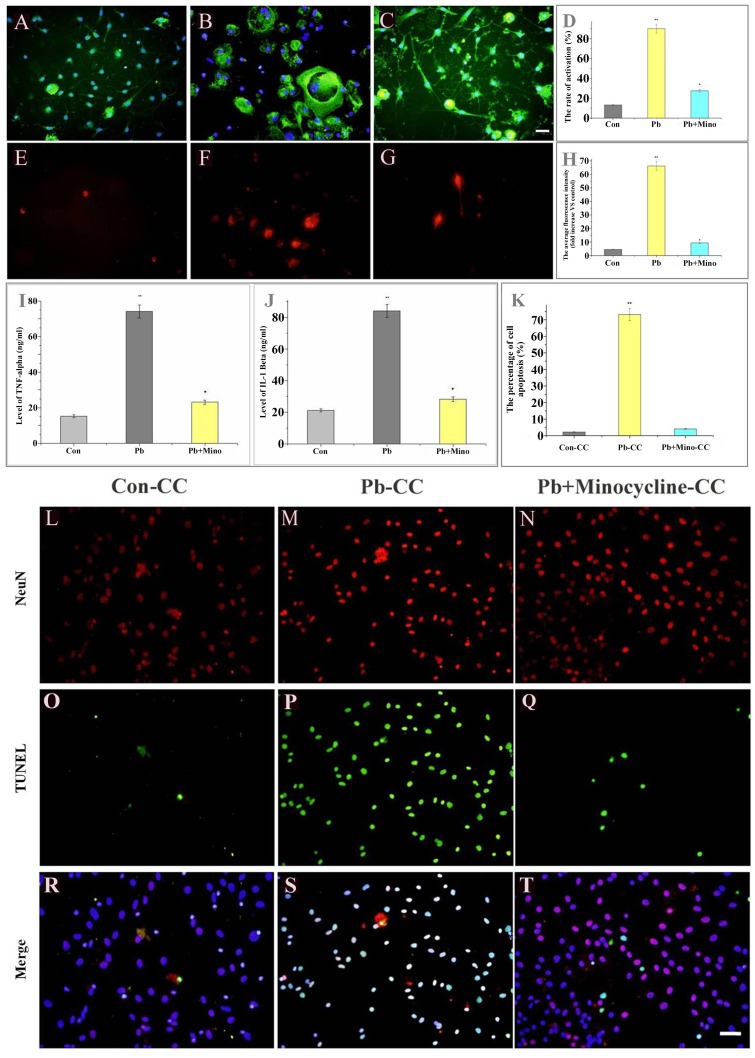
Minocycline blocks the activation of microglia; attenuates Pb-induced secrete of TNF-α, IL-1β and expression of iNOS; and protects co-cultured hippocampal neurons. Purified microglia were treated with vehicle (control) (A), Pb (50 µmol) (B), or Pb (50 µmol)+minocycline (45 µmol) (C) for 48 h, and were co-cultured with hippocampal neurons for another 48 h. (A, B, C) Microglia activation was detected by OX-42 antibody. (D) The results were quantified. Results (A, B and C) are expressed as the mean ± S.D. of average activated cell rate in random fields (n = 20). **P<0.001 compared with control groups. *P<0.01 compared with Pb-treated groups. iNOS was detected by anti-iNOS antibody (E, F, G. red fluorescence). Activation of iNOS was evaluated by measure the average fluorescence of iNOS-immunoreactive cells. (H) The results were quantified. Results (E, F and G) are expressed as the mean ± S.D. of average fluorescence of iNOS-immunoreactive cell number in random fields (n = 20). **P<0.001 compared with control groups. *P<0.01 compared with Pb-treated groups. TNF-α (I) and IL-1β (J) were determined by ELISA. Purified microglia were treated with vehicle (control) or Pb (50 µmol), or Pb (50 µmol)+minocycline (45 µmol) for 48 h for the TNF-α and IL-1β assay, respectively. **P<0.001 compared with control groups. *P<0.01 compared with Pb-treated groups. Apoptotic hippocampal neurons were detected and compared using TUNEL (green fluorescence) (L–T). Degeneration of hippocampal neurons was evaluated by counting the number of apoptotic neurons. (K) The results were quantified and are expressed as the mean ± S.D. of apoptotic neurons percentage in random fields (n = 20). **P<0.001 compared with control groups. *P<0.01 compared with Pb-treated groups. All scale bar indicates 50 µm.

Minocycline, a tetracycline antibiotic, has profound anti-inflammatory properties and is used experimentally for the treatment of many central nervous system (CNS) disorders. Minocycline has been shown to inhibit microglia activation, and to protect the CNS under inflammatory conditions [29], [30], [31]. In order to establish whether secretions from activated microglia caused hippocampal neuronal death, cultures were treated with 50 µM Pb and 45 µM minocycline. The results showed that minocycline strongly inhibited microglia activation (Con: 13.34±0.67%, n = 20; Pb: 90.13±4.51%, n = 20; Minocycline: 27.67±1.38%, n = 20) (Fig. 4 A–D), meanwhile, decreased iNOS (Con: 4.46±0.22, n = 20; Pb: 66.10±3.30, n = 20; Minocycline: 9.32±0.47, n = 20) (Fig. 4 E–H), TNF-α (Con: 15.22±0.76%, n = 6; Pb: 73.76±3.37%, n = 6; Minocycline: 23.04±1.10%, n = 6) (Fig. 4 I) and IL-1β (Con: 21.12±1.23%, n = 6; Pb: 84.20±3.70%, n = 6; Minocycline: 28.39±1.36%, n = 6) (Fig. 4 J) expression.

### *In vitro* pretreatment with minocycline prevents hippocampal neuronal injury

**Figure 5:**
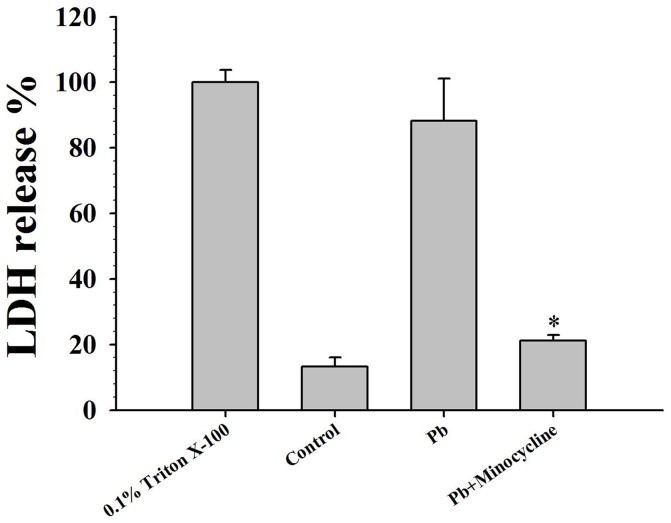
Effect of minocycline on LDH-release after Pb exposure. Purified microglia were treated with vehicle (control), Pb (50 µmol) or Pb (50 µmol)+minocycline (45 µmol) for 48 h, and were co-cultured with hippocampal neurons for another 48 h. LDH-release were measured from co-culture supernatant by LDH-cytotoxicity assay kit. The mean value of LDH-release of the positive control group (0.1% triton X-100) was considered as 100%. n = 6. Mean ± S.D. *P<0.01 compared with Pb-treated groups.

We confirmed that minocycline inhibited microglia activation and the expression of their inflammatory factors. However, does this inhibition lead to the prevention of hippocampal neuronal death? Minocycline pretreated microglia were co-cultured with hippocampal neurons, and number of apoptotic neurons were significantly less (4.11±0.21%, n = 20) (Fig. 4 K, N, Q and T) than that of cells pretreated with 50 µM Pb alone (73.15±3.66%, n = 20) (Fig. 4 K, M, P and S). On the other hand, we also confirmed that after Pb treatment, the average value of LDH was similar to that of the positive control group treated with 0.1% Triton X-100 (88.17±12.92%, n = 6, *P*>0.05 VS positive control) (Fig. 5). When minocycline was added to the cultures during Pb exposure, the average value of LDH greatly declined (21.21±1.63%, n = 6) (Fig. 5) as compared with Pb-treated groups (*P*<0.01). These suggested that minocycline could prevent Pb-induced hippocampal neuronal injury.

### Minocycline pretreatment reversed Pb-induced inhibition of hippocampal LTP

**Figure 6:**
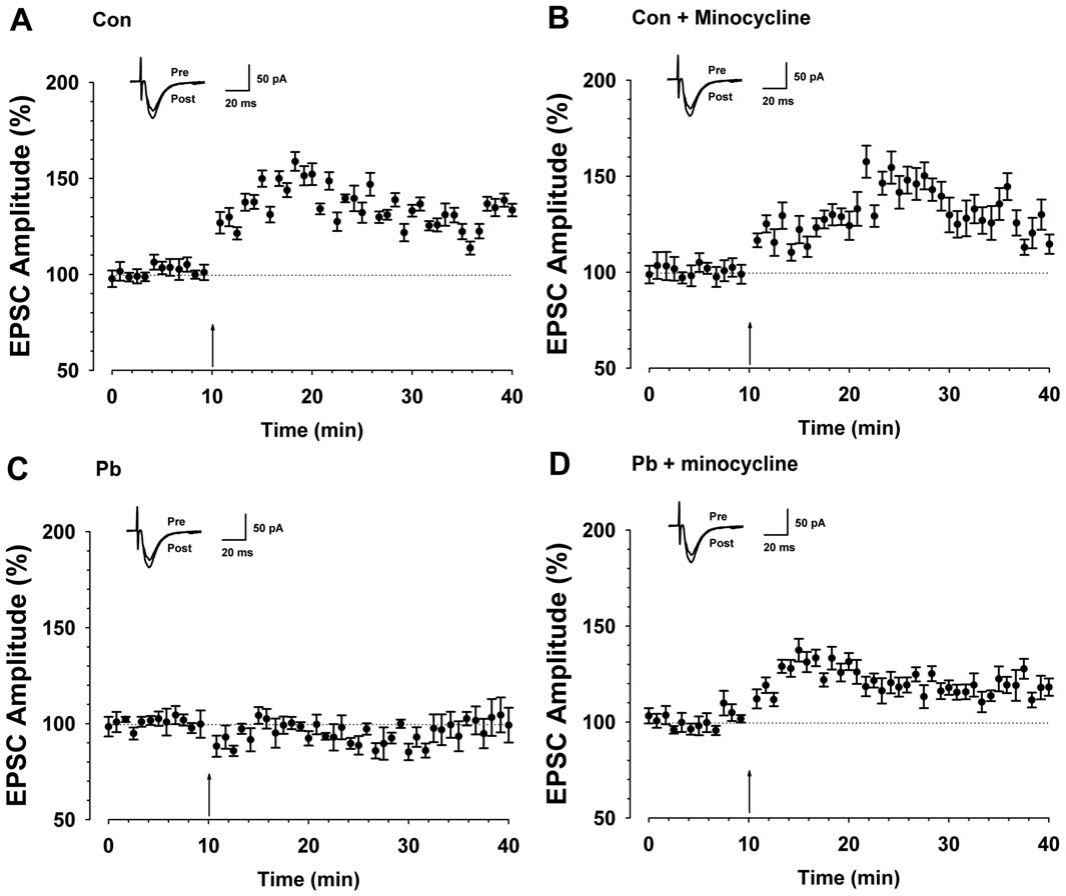
Minocycline reversed the induction of LTP in hippocampus after 100 ppm Pb treated. (A) LTP was induced in hippocampal pyramidal neurons in control rats (n = 11 slices/6 rats, t-test; *P*<0.001 compared with baseline). (B) LTP was induced in hippocampal pyramidal neurons in minocycline treated rats (n = 8 slices/6 rats, t-test; *P*<0.001 compared with baseline). (C) LTP was lost in hippocampal pyramidal neurons in Pb treated rats (n = 10 slices/6 rats, t-test; *P*>0.05 compared with baseline). (D) LTP was partly reversed in hippocampal pyramidal neurons in minocycline treated rats during Pb exposure (n = 9 slices/6 rats, t-test; *P*<0.05 compared with baseline). Pairing training is indicated by an arrow. The dashed line indicates the mean basal synaptic responses.

We confirmed that pretreatment with minocycline partly reversed Pb-induced activation of microglia and hippocampal neuronal injury *in vitro*. The study of the LTP in hippocampus *in vivo* also showed that, after minocycline administration, the induction of LTP (121.2±4.8%, n = 9 slices/6 rats, t-test; *P*<0.05 compared with baseline, Fig. 6D) were partly reversed compared with Pb treated rats (95.4±5.3%, n = 10 slices/6 rats, t-test; *P*>0.05 compared with baseline, Fig. 6C). The results also showed that there is no significant different between control rats (135.1±4.1% of baseline, n = 11 slices/6 rats, t-test; *P*<0.001 compared with baseline, Fig. 6A) and minocycline treated rats (130.7±6.7% of baseline, n = 8 slices/6 rats, t-test; *P*<0.001 compared with baseline, Fig. 6B). This suggested that minocycline could partly prevent Pb-induced LTP impairment.

## Discussion

In the present study, we revealed that low-level Pb exposure can activate microglia and increase the release of some cytokines such as IL-1β and TNF-α and up-regulate the expression of iNOS, meanwhile, such changes were accompanied with hippocampal neuronal injury and hippocampal LTP impairment. Inhibiting Pb-induced microglia activation with minocycline also reversed Pb enhanced cytokine release as well as hippocampal neuronal injury and LTP impairment. This might be the underlying mechanisms that Pb impairs the hippocampus dependent learning and memory.

Pb toxicity on neurons but not glia cells is commonly taken into consideration when investigating the Pb-induced LTP and learning and memory impairments [32], [33]. However, microglial cells play very important roles in varied physiological or pathophysiological functions [34], [35]. They are the only nucleus phagocytes within CNS which are very important to the CNS immune responses [36]. The most remarkable feature of microglia is their extreme sensitivity to external environmental stimuli [36]. Moreover, activated microglia can release many cytokines, including TNF-α, IL-1β and interferon gamma (IFN-γ), and they also up-regulate the expression of iNOS. These factors are believed to be the main causes of neurotoxicity [27]. Treatment with some neurotoxic substance such as LPS impairs the rat learning and memory [26], and induces the microglia activation [37]. Pb is also a neurotoxic substance, therefore, it is natural to speculate that microglia may play an important role in the Pb-exposure induced learning and memory decline. In consistent with our hypothesis, we did detect Pb-induced microglia activation accompanied with the long-term potentiation impairment. Moreover, within our expectation, all these changes were also accompanied with hippocampal neuronal injury (by *in situ* TUNEL and LDH release assay).

Pb-exposure can impair the hippocampal LTP and such impairment is suggested to be mediated by the alteration of glutamate-N-methyl-D-aspartate (NMDA) receptor system [38]. These findings fell into the conventional neuron-centered theory. We moved forward and revealed the substantial involvement of microglia in the Pb-induced hippocampal LTP impairment. Furthermore, such effects of microglia might be mediated by the released cytokines. But it is not elucidated whether these effects are direct or indirect. By using microglia and hippocampal neurons co-culture model, we offered the evidence that microglia affects the neuronal functions. By treating rats with minocycline we evidence that microglia impair the hippocampal LTP. In other word, microglia affects hippocampal LTP and learning and memory indirectly. However, the possibility of direct effects can not be ruled out, as other studies demonstrated such evidence [39], [40].

In this study, we treated microglia with 50 µmol Pb based on our MTT assay results. Although this dose is well beyond USA CDC stated safe limit, we reported these data to demonstrate that in *in vitro* experiment, this dose Pb exposure results in robust activation of microglia and cytokines release, which may contribute to the primary cultured hippocampal neuronal injury.

In summary, our current study suggests that Pb neurotoxicity may be mediated by microglia activation, which induces high-level expression of many factors. Either alone or in combination, these inflammatory factors may cause hippocampal neuronal injury. This neurotoxic damage may affect the induce level of LTP, thus leading to learning and memory deficits.

## Supporting Information

Figure S1 SD-rat blood leads level. SD-rat were treated with 0 (control) or 100, 200, 300 ppm lead acetate for 8 weeks from drink water. Data are expressed as mean ± SD (n = 6).
(TIF)

Figure S2 SD-rat hippocampus lead level after treated with lead acetate for eight weeks. SD-rat were treated with 0 (control) or 100, 200, 300 ppm lead acetate for 8 weeks from drink water. Data are expressed as mean ± SD (n = 6).
(TIF)

Figure S3 SD-rat body weight. SD-rat were treated with 0 (control) or 100, 200, 300 ppm lead acetate for 8 weeks. Data are expressed as mean ± SD (n = 20).
(TIF)

Figure S4 SD-rat water consumption after treated with lead acetate. SD-rat were treated with 0 (control) or 100, 200, 300 ppm lead acetate for 8 weeks. Data are expressed as mean ± SD (n = 40).
(TIF)

Figure S5 Effect of lead on primary cultured microglia by MTT assay. Primary cultured microglia were treated with 0, 25, 50, 100, 200, 300, 400, 500, 600 µmol lead acetate for 0, 3, 6, 9, 12, 24 and 48 hours. All data are expressed as mean ± SD (n = 6 well).
(TIF)
