# Supplementary figures and images for: Involvement of Microglia Activation in the Lead Induced Long-Term Potentiation Impairment
Source: PLoS One. 2012 Aug 31;7(8):e43924. doi: 10.1371/journal.pone.0043924 (PMC3432044; doi:10.1371/journal.pone.0043924)

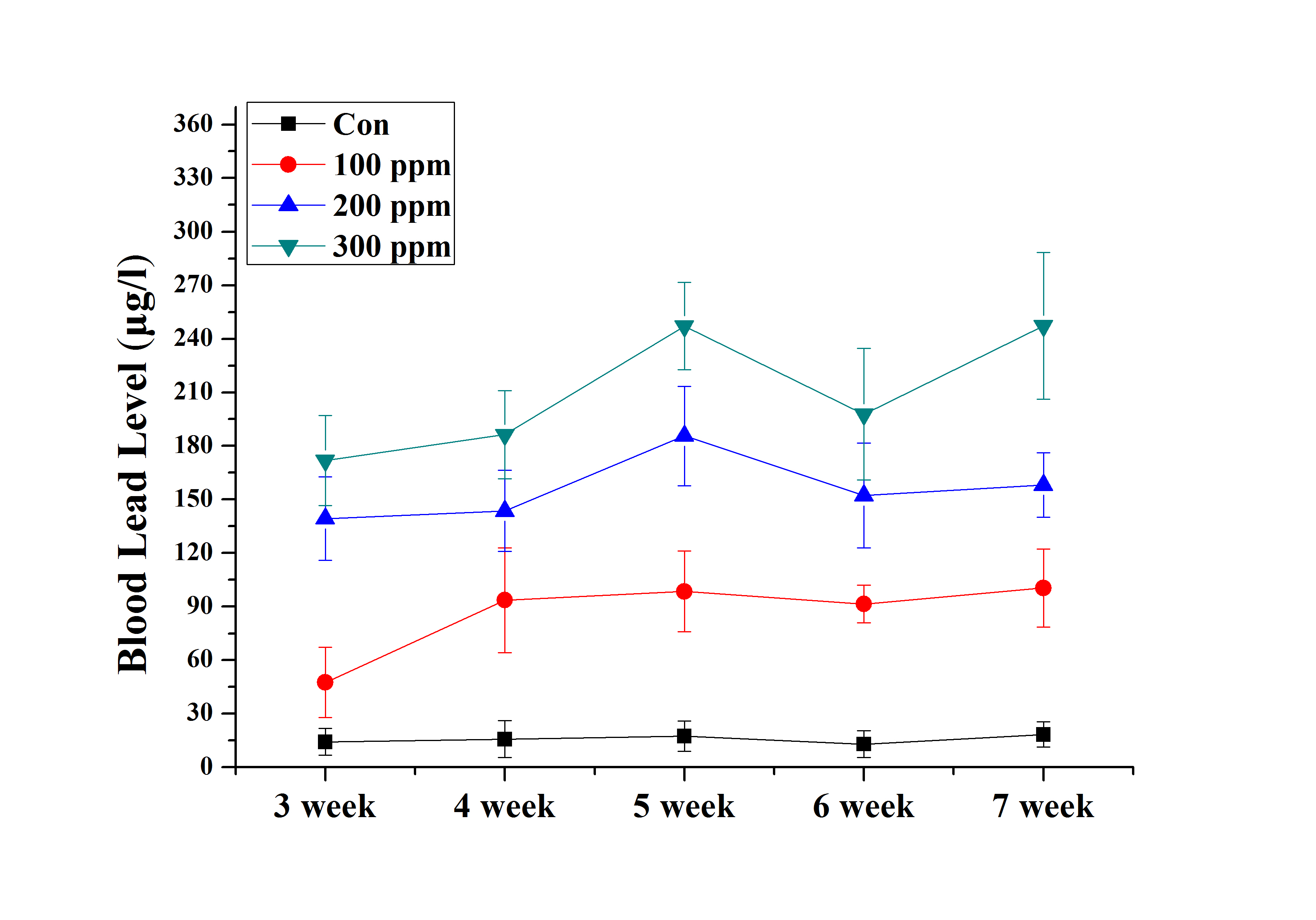

Supplement: Figure S1 — SD-rat blood leads level. SD-rat were treated with 0 (control) or 100, 200, 300 ppm lead acetate for 8 weeks from drink water. Data are expressed as mean ± SD (n = 6). (TIF) [file pone.0043924.s001.tif]

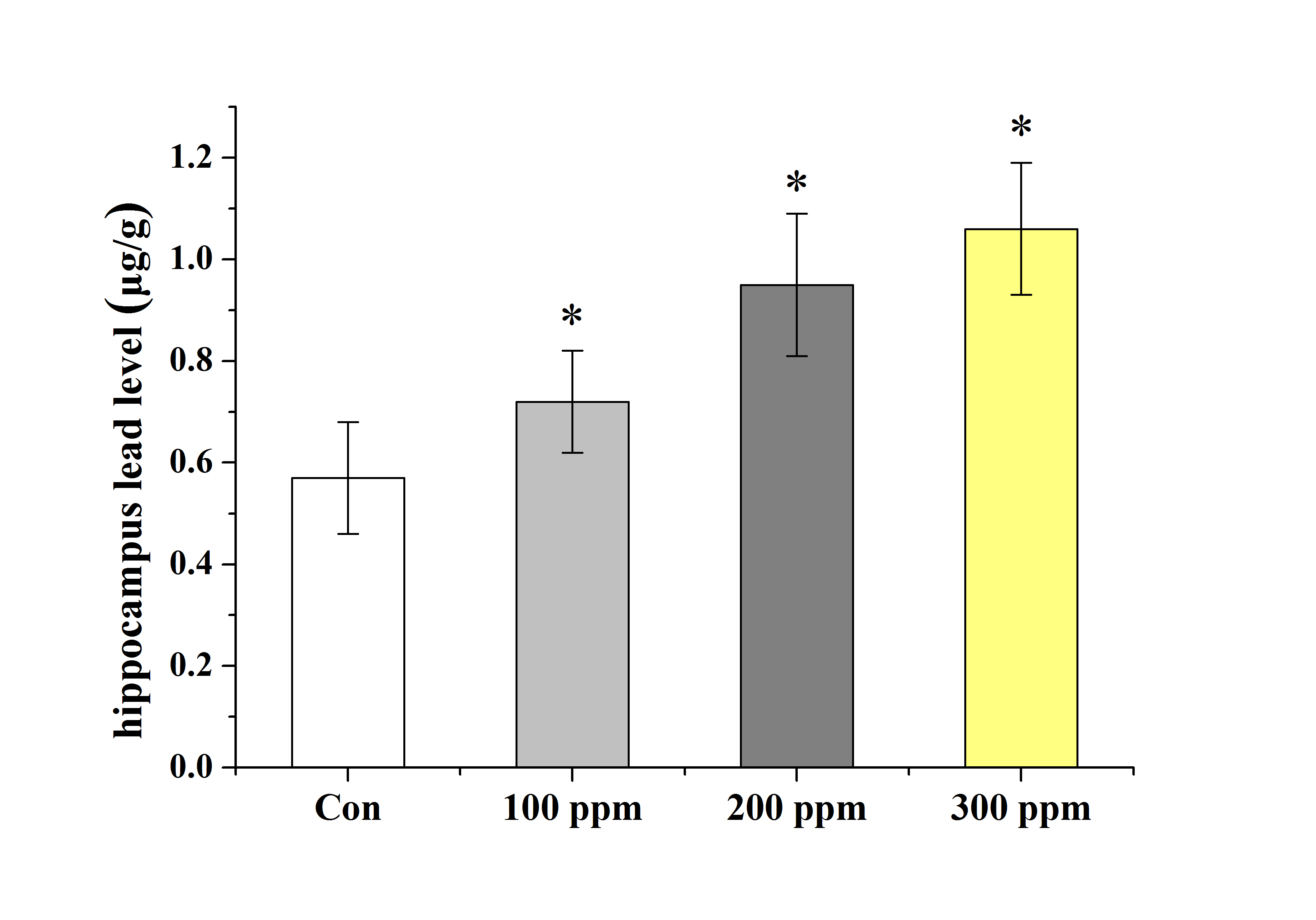

Supplement: Figure S2 — SD-rat hippocampus lead level after treated with lead acetate for eight weeks. SD-rat were treated with 0 (control) or 100, 200, 300 ppm lead acetate for 8 weeks from drink water. Data are expressed as mean ± SD (n = 6). (TIF) [file pone.0043924.s002.tif]

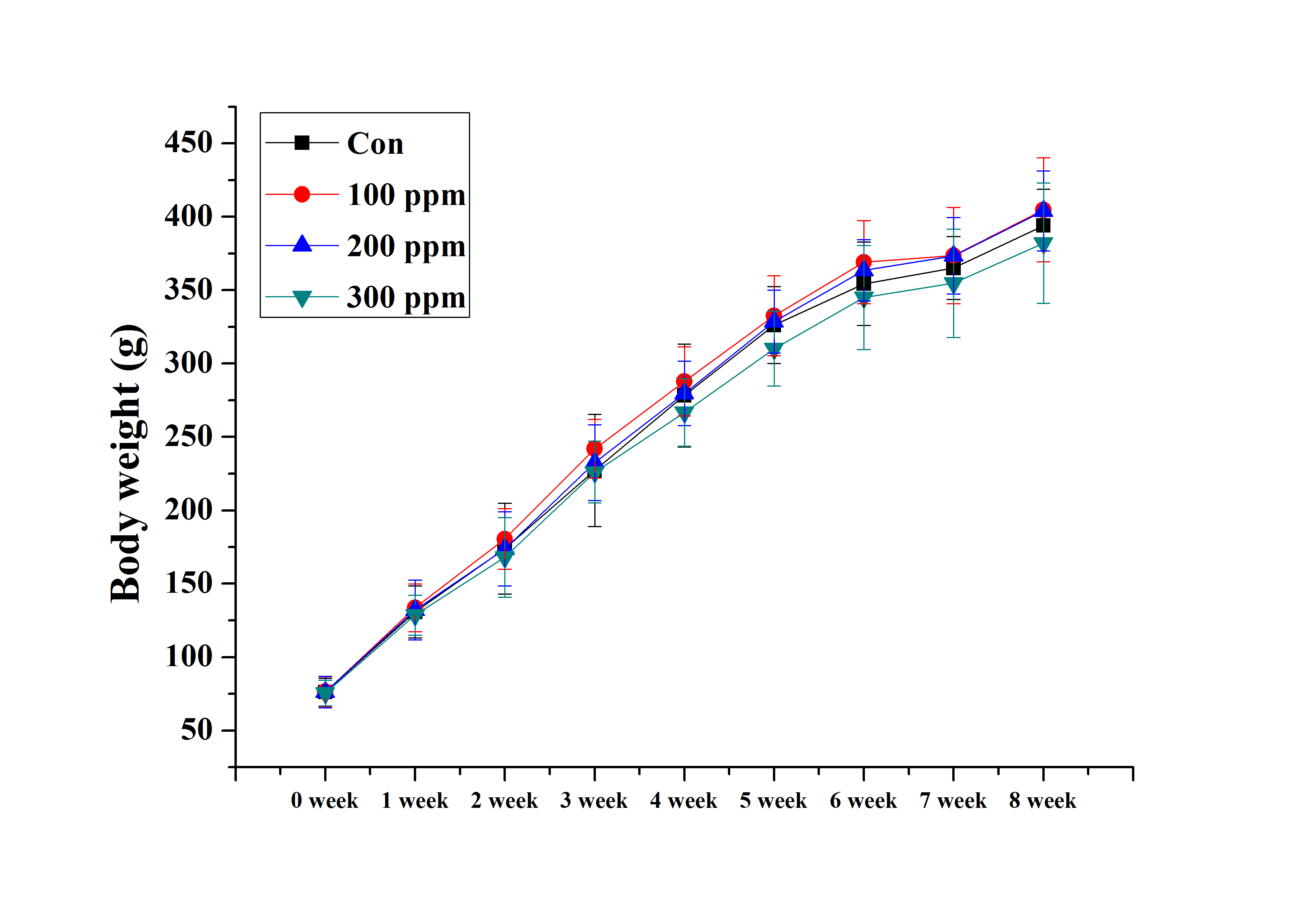

Supplement: Figure S3 — SD-rat body weight. SD-rat were treated with 0 (control) or 100, 200, 300 ppm lead acetate for 8 weeks. Data are expressed as mean ± SD (n = 20). (TIF) [file pone.0043924.s003.tif]

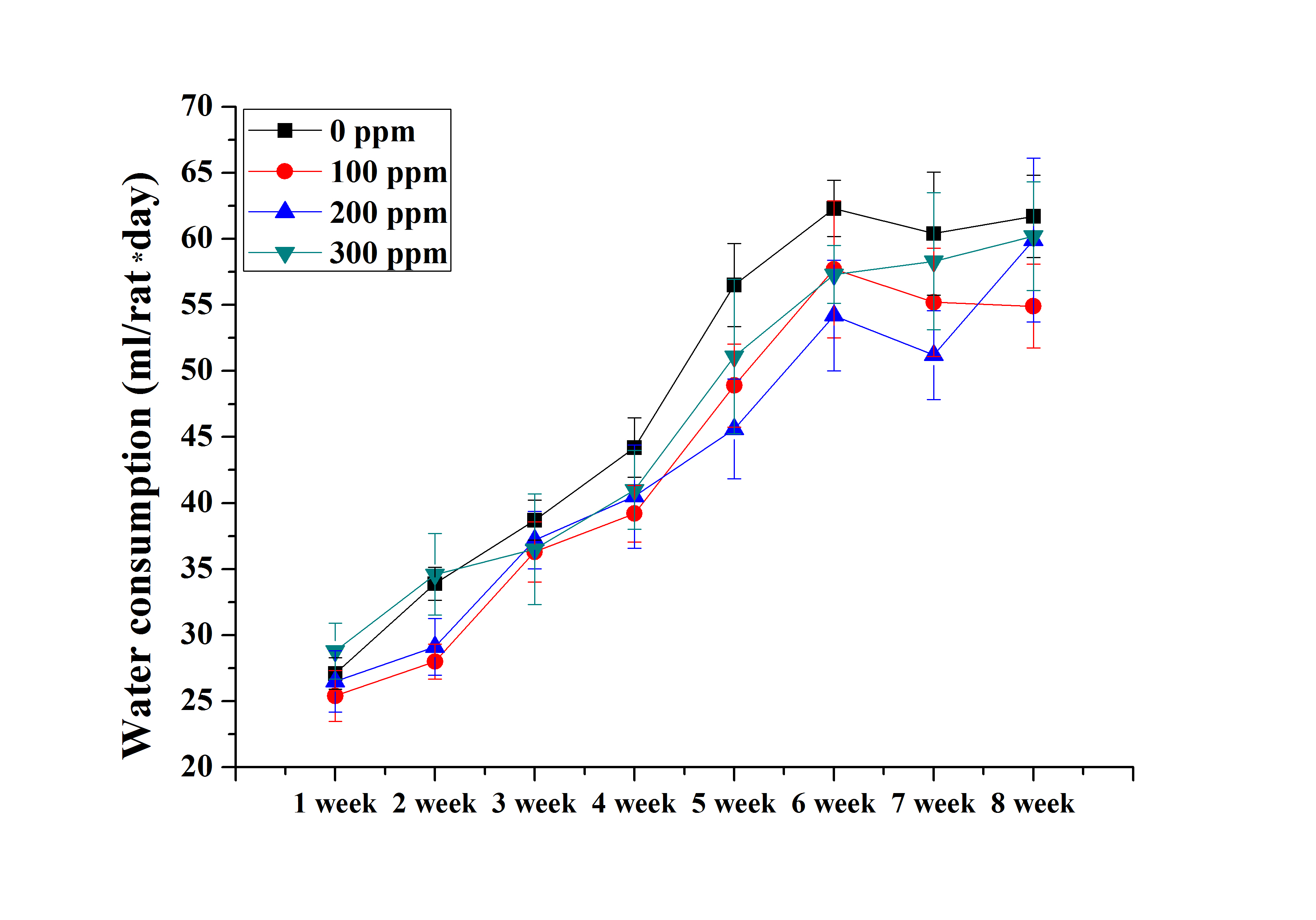

Supplement: Figure S4 — SD-rat water consumption after treated with lead acetate. SD-rat were treated with 0 (control) or 100, 200, 300 ppm lead acetate for 8 weeks. Data are expressed as mean ± SD (n = 40). (TIF) [file pone.0043924.s004.tif]

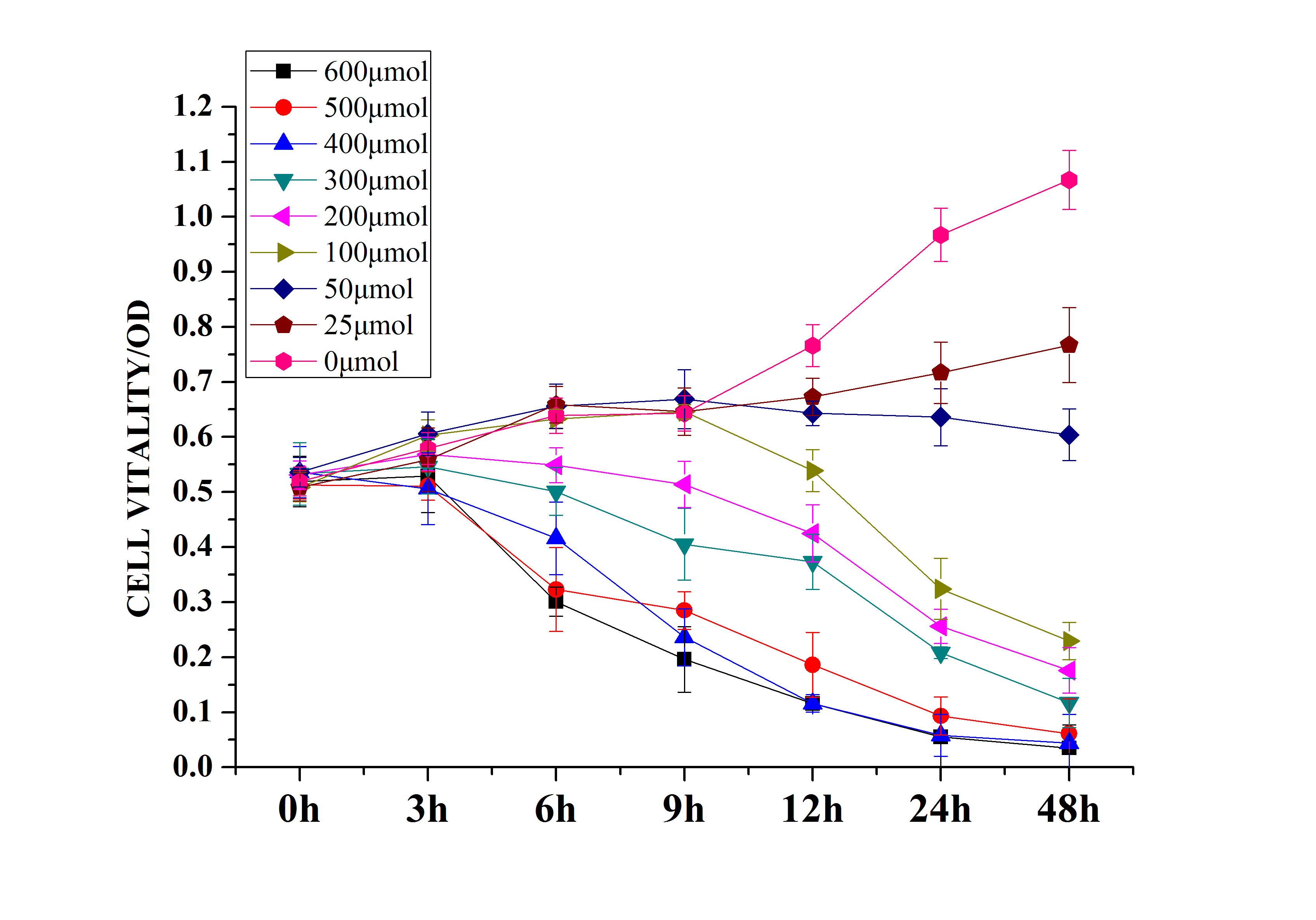

Supplement: Figure S5 — Effect of lead on primary cultured microglia by MTT assay. Primary cultured microglia were treated with 0, 25, 50, 100, 200, 300, 400, 500, 600 µmol lead acetate for 0, 3, 6, 9, 12, 24 and 48 hours. All data are expressed as mean ± SD (n = 6 well). (TIF) [file pone.0043924.s005.tif]
